# Modelling Lymphoma Therapy and Outcome

**DOI:** 10.1007/s11538-013-9925-3

**Published:** 2013-12-14

**Authors:** Katja Roesch, Dirk Hasenclever, Markus Scholz

**Affiliations:** 1 Institute for Medical Informatics, Statistics and Epidemiology, University of Leipzig, Härtelstraße 16-18, 04107 Leipzig, Germany; 2 LIFE Research Center for Civilization Diseases, University of Leipzig, Philipp-Rosenthal-Straße 27, 04103 Leipzig, Germany

**Keywords:** Chemotherapy, Differential equation based model, Immune system, Lymphoma, Survival analysis

## Abstract

Dose and time intensifications of chemotherapy improved the outcome of lymphoma therapy. However, recent study results show that too intense therapies can result in inferior tumour control. We hypothesise that the immune system plays a key role in controlling residual tumour cells after treatment. More intense therapies result in a stronger depletion of immune cells allowing an early re-growth of the tumour.

We propose a differential equations model of the dynamics and interactions of tumour and immune cells under chemotherapy. Major model features are an exponential tumour growth, a modulation of the production of effector cells by the presence of the tumour (immunogenicity), and mutual destruction of tumour and immune cells. Chemotherapy causes damage to both, immune and tumour cells. Growth rate, chemosensitivity, immunogenicity, and initial size of the tumour are assumed to be patient-specific, resulting in heterogeneity regarding therapy outcome. Maximum-entropy distributions of these parameters were estimated on the basis of clinical survival data. The resulting model can explain the outcome of five different chemotherapeutic regimens and corresponding hazard-ratios.

We conclude that our model explains observed paradox effects in lymphoma therapy by the simple assumption of a relevant anti-tumour effect of the immune system. Heterogeneity of therapy outcomes can be explained by distributions of model parameters, which can be estimated on the basis of clinical survival data. We demonstrate how the model can be used to make predictions regarding yet untested therapy options.

## Introduction

### Medical and Biological Background

**Fig. 1:**
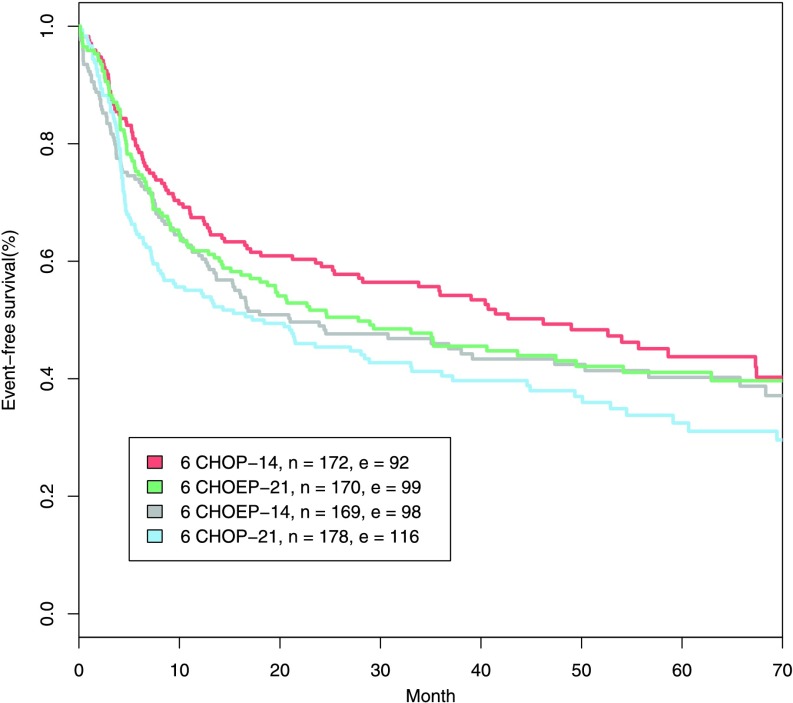
Event-free survival of older patients from NHL-B2. Standard CHOP-21 chemotherapy was compared with dose-intensification (adding of the additional drug etoposide (CHOEP-21)), time-intensification (shortening the cycle duration (CHOP-14)) and double intensification (CHOEP-14). While the first two intensifications are superior, the double intensification does not result in further improvements. *n* and *e* indicate the number of patients and the number of events

High-grade non-Hodgkin lymphoma (NHL) is a haematologic malignancy, which is curable by multi-drug and multi-cycle cytotoxic chemotherapy in a substantial proportion of cases (DeVita et al. 1975). The classical model of chemotherapy action and tumour regrowth proposed by Skipper et al. (1970) provided a rationale for chemotherapy intensifications either by increasing the dose of the drugs or the number of cycles, adding additional drugs or shortening the time between chemotherapy cycles. Accordingly, a number of trials were performed which, however, were only partially successful. For example, in the NHL-B2 trial (Pfreundschuh et al. 2004a), it has been shown that either dose- or time-intensification of the standard multi-drug chemotherapy containing cyclophosphamide, doxorubicin, vincristine, and prednisone (CHOP) improves the outcome. But double-intensification is inferior, which cannot be explained away by increased toxic side-effects (see Fig. 1). Since this phenomenon partially disproves the Skipper paradigm, we aim at extending this model. We hypothesise that the immune system plays a crucial role in controlling residual tumour cells and treatment effects on the immune system need to be considered.

There is some biological evidence supporting a role of the immune system: The immune system responds to a growing tumour in a cell-mediated way involving predominantly cytotoxic T-lymphocytes and natural killer cells. It is well known that cancer cells are attacked and killed by these immune cells. This is also the case for lymphoma and especially NHL. For instance, patients with rheumatoid arthritis treated with immune-suppressing methotrexate have a higher risk in developing lymphoma. After the withdrawal of methotrexate, the lymphoma vanishes in some cases (Lim and Bertouch 1988; Mariette et al. 2002; Niitsu et al. 2010; Wang et al. 2010). Lymphoma sometimes occurs in patients under immunosuppression, e.g. after bone marrow transplantation. These lymphomas can regress spontaneously after recovery of the immune system (Mohsin et al. 2007; Nalesnik et al. 1988). The so-called graft-versus-lymphoma effect after allogeneic haematopoietic stem-cell transplantation is also associated with the effect of immune cells on tumour cells, i.e. immune cells of the donor attack tumour cells of the host (Bishop et al. 2008; Dodero et al. 2012). Patients suffering from AIDS have a 165-fold increased risk to get NHL than persons without AIDS (Coté et al. 1997). Finally, there are cases of spontaneous regression of NHL without apparent reasons, which could be explained by immune control (Abe et al. 2007; Cole 1974; Engel and Lee 2009; Iwatani et al. 2011; Watari et al. 2005).

Massive doses of chemotherapy, too many cycles, or too few days between dose applications result in a strong depletion of immune cells. In consequence, remaining immune cells may transiently be unable to control residual tumour cells responsible for relapse in a critical period just after the end of therapy. This might explain paradoxic effects of therapy intensifications observed in clinical trials. Therefore, we propose a model of the dynamics of tumour, immune system, and their interactions considering the effects of chemotherapy and patient heterogeneity.

The optimal choice of dose and time schedule in chemotherapy is an important and intricate question. We are in particular interested in describing and understanding these phenomena by model simulations. For this purpose, it is necessary to parametrise the model for humans. Outcome data is available as event-free survival curves derived from clinical trials. Since these data only provide limited information regarding the dynamics of the tumour and immune system, the model is kept as simple as possible in order to reduce the number of free parameters.

In the present paper we explain our model assumptions, requirements and equations, derive the mathematical properties of the model and describe a technique for parameterising the model on the basis of clinical survival data. We discuss the plausibility of our model on parameters in detail. Finally, we demonstrate how the model can be used to make predictions regarding yet untested therapy options.

### Model Assumptions and Requirements

We propose an ordinary differential equations (ODE) model of the interaction of a growing tumour, an anti-tumour immune response, and the effect of chemotherapy on both the tumour and immune system. The system is based on a model proposed by Kuznetsov et al. (1994). To keep our model simple, the immune system will be represented by a single equation describing the overall status of the immune cell population. This population is called *effector cells* in the following and fits best to the CD8+ T-cell population. We construct a model of the dynamics of tumour and effector cell populations, i.e. we consider large numbers of cells rather than single-cell interactions. The model is based on the following mechanistic assumptions and requirements:

1. The tumour grows uncontrolled in the absence of chemotherapy and immune effector cells.
2. Both effector cells and chemotherapy reduce the number of tumour cells.
3. The production of tumour-specific effector cells is stimulated by the presence of tumour cells (immunogenicity of the tumour).
4. There is a loss of effector cells due to degradation, consumption by fighting the tumour, and chemotherapy.
5. Higher chemotherapy doses result in both higher tumour and effector cell depletion.

The model will be linked to clinical survival data derived from randomised studies, with the aim to predict and explain data of chemotherapy outcomes. More precisely, we claim that the model fulfils the following requirements:

1. heterogeneity of patient outcomes can be traced back to plausible heterogeneity of certain model parameters,
2. the estimated parameters and parameter distributions are biologically plausible,
3. the time scale and variance of time to relapse is consistent with reported event-free survival curves,
4. extent of tumour at diagnosis (e.g. stage) emerges as prognostic factor,
5. observed treatment difference of several clinical trials comparing CHOP and CHOEP variants are quantitatively reproduced.

In particular, we are interested in exhibiting the possibility of “paradoxic” treatment effects namely that a more intense chemotherapy has not superior, but even inferior results. This may happen if chemotherapy is not able to completely eradicate the tumour but the immune system is weakened to such an extent that it cannot control residual tumour cells.

## The ODE-Model for Immunogenic Lymphoma

As basic model of an immunogenic tumour we adapt a model of Kuznetsov et al. (1994) originally developed for leukaemia in mice. For this purpose, we simplify model equations, retrieve parameters valid for humans and take into account that lymphoma grow in compact nodes. Some of the model parameters are determined on the basis of biological reasoning. Others may vary between patients. The distribution describing this heterogeneity will be estimated later using clinical survival data (Sect. 3). For qualitative model analysis, we used the parameters proposed by Kuznetsov et al. (1994) (Sect. 2.1).

Additionally, the effect of chemotherapy is added as transient depletion of cell counts following a first order kinetic (i.e. a fixed percentage of cells survive) both for tumour and for immune effector cells. Corresponding parameters require estimation, also (Sect. 3).

### Kuznetsov’s Model for Immunogenic Tumours

The ODE-model introduced by Kuznetsov is based on a system of two ordinary differential equations describing the kinetics of growth and regression of an immunogenic tumour and the interaction with tumour-specific effector cells of the immune system. The model exhibits two major phenomena that were observed in experiments with BCL_1_-mice: Firstly, tumour growth stimulates the production of new cytotoxic T-lymphocytes; secondly immune cells destroy tumour cells. Depending on parameter settings, possible stable outcomes of the models are extinction or (saturated) permanent growth of the tumour or persistence of a residual tumour controlled by the immune system. This model meets all of our assumptions and requirements made in Sect. 1.2 except for the incorporation of chemotherapy.

In the following, let *T* be the number of tumour cells and *E* be the number of tumour-specific effector cells. The system of differential equations of the model in Kuznetsov et al. (1994) reads

1 $$\begin{aligned} \begin{aligned}[c] \frac{dE}{dt} &= \sigma+ \frac{\rho ET}{\eta+T}-\mu ET- \delta E, \\ \frac{dT}{dt} &= \alpha T(1-\beta T)- \nu ET. \end{aligned} \end{aligned}$$

The parameters have the following biological interpretations:

*σ*: tumour-independent production rate of effector cells,
*ρ*: tumour-induced rate of effector cell stimulation,
*η*: the number of tumour cells where the stimulation of effector cells is half-maximal (smaller *η* means more rapid increase of effector cell stimulation),
*μ*: tumour-induced rate of effector cell inactivation,
*δ*: rate of tumour-independent effector cell inactivation,
*α*: maximal rate of tumour growth,
*β*: inverse of tumour carrying capacity,
*ν*: rate of tumour cell elimination induced by effector cells.

Kuznetsov estimated the parameters of the model on the basis of experimental data in mice (Kuznetsov et al. 1994, pp. 302–303, see Table 1). For this set of parameters, the ODE system has four steady states:

(A) *T*=0, $E= \frac{\sigma}{\delta} \approx3.2\cdot10^{5}$ (tumour-free effector cell population).
(B) *T*≈4.6⋅10^8^, *E*≈1.6⋅10^5^ (large tumour).
(C) *T*≈8⋅10^6^, *E*≈1.6⋅10^6^ (“dormant” tumour).
(D) *T*≈2.5⋅10^8^, *E*≈8⋅10^5^.

**Table 1:** Overview of the model parameters. We present values for constant parameters and ranges for parameters assumed to be heterogeneous

Quality	Human value	Kuznetsov’s value	Unit	Interpretation	Reference for human values
*σ*	1400	1.3⋅10^4^	cells⋅day^−1^	tumour-independent production rate of effector cells	Alanio et al. (2010), Moon et al. (2007)
*ρ*	[0.01,0.21]	const. (0.1245)	day^−1^	tumour-induced stimulation rate of effector cells	
*η*	2.019⋅10^5^	2.019⋅10^7^	cells	number of tumour cells where effector cell stimulation rate is half-maximal	
*μ*	3.422⋅10^−10^	equal	day^−1^ cells^−1^	tumour-induced inactivation rate of effector cells	Kuznetsov et al. (1994), Ladell et al. (2008)
*δ*	0.007	0.0412	day^−1^	inactivation rate of effector cells	
*α*	[0.01,0.5]	const. (0.18)	day^−1^	tumour growth rate	
*β*	0	2⋅10^−9^	cells^−1^	inverse tumour-carrying capacity	
*ν*	1.101⋅10^−7^	equal	day^−1^ cells^−1^	effector induced elimination rate of tumour cells	Kuznetsov et al. (1994)
*c*	0.75	1		exponent in interaction terms of effector and tumour cells, corresponding to dim. of tumour surface	
*E*_equ_	2⋅10^5^	3.2⋅10^5^	cells	tumour-free equilibrium state of effector cells	Alanio et al. (2010), Moon et al. (2007)
*T*_diag_	[10^10^,10^13^]	x	cells	tumour size at diagnose	
*T*_rez_	10^11^	x	cells	tumour size at relapse	
*k*_*T*_	[0.01,9.21]	x	day^−1^	tumour log cell kill due to chemotherapy	
*k*_*E*_	[0.01,1.6]	x	day^−1^	effector log cell kill due to chemotherapy	

The steady state (D) is a saddle node on a curve, the separatrix, which divides the phase space into two domains: Initial conditions at one domain result in uncontrolled tumour growth (stable steady state (B)) while at the second domain the tumour is eventually controlled by the immune system (stable steady state (C)). Steady state (A) is an unstable fixed point and expresses a tumour-free effector cell population. The number of fixed points as well as their stability depend on the parameter setting.

Kuznetsov’s model is designed to express the interactions of tumour cells and immune cells in leukaemia for mice. We adapt this ODE-system to model tumour growth and therapy in human diffuse large B-cell lymphoma (DLBCL).

### ODE-Model for Immunogenic Diffuse Large B-Cell Lymphoma in Humans

In Kuznetsov’s model, the terms representing interaction of effector and tumour cells are linear in both. This is not adequate for large cell lymphoma as the contact of tumour and immune system is limited mainly to the tumour surface. Thus, the interaction of effector cells and tumour is proportional to the tumour surface which is proportional to *T* ^2/3^ by the following considerations: We treat the tumour as a sphere. Then the number of cells *T* is proportional to its volume: *T*∝*r* ^3^ where *r* denotes the radius of the tumour. The tumour surface is proportional to *r* ^2^∝*T* ^2/3^. As B-cell-lymphomas grow in multiple tumour nodes, we set the exponent slightly larger than $\frac{2}{3}$. In the following, we denote it as *c* and set *c*=0.75 (see also Table 1):

$$ E \propto T^{c},\quad c= 0.75. $$

Kuznetsov defines $\frac{1}{\beta} = 5\cdot10^{8}$ for the carrying capacity of the tumour. The human body comprises a cell number of the order of 10^14^ (Alberts et al. 2002). Thus, we estimate that Human B-cell lymphomas cannot exceed a size of 10^14^, resulting in *β*≈10^−14^. However, in order to estimate relapse times after therapy, the simulation of our model is stopped if a tumour cell number of about 10^13^ is exceeded. Since saturation is not achieved at this cell count, we can assume *β*≈0 for the purpose of treatment evaluation. This assumption implies an exponential tumour growth in the absence of immune controls. It appears to be justified for tumours in humans at time of diagnosis (see, e.g. Norton and Simon 1979; Mackillop 1990).

The modified differential equation (1) of interaction between effector and tumour cells now reads

2 $$\begin{aligned} \begin{aligned}[c] \frac{dE}{dt} &= \sigma+ \frac{\rho ET^c}{\eta+T^c}-\mu ET^c- \delta E, \\ \frac{dT}{dt} &= \alpha T- \nu ET^c. \end{aligned} \end{aligned}$$

According to Skipper et al. (1970), we assume that a specific percentage of tumour is destroyed in each therapy cycle. We constructed biomathematical models of haematopoiesis under chemotherapy in the past showing that the loss of effector cells can also be explained by the destruction of a specific percentage of these cells in each chemotherapy cycle (Scholz et al. 2005). Hence, we added a first order loss term in our ODE model equations for the duration of one day after chemotherapy. The magnitude of reduction can be controlled by the parameters *k*_*T*_ for tumour cells and *k*_*E*_ for effector cells:

$$\begin{aligned} &\frac{dE}{dt} = \sigma+ \frac{\rho ET^c}{\eta+T^c}-\mu ET^c- \delta E- k_EE\mathbf{1}_{\mathbf{CT}}, \\ &\frac{dT}{dt} = \alpha T- \nu ET^c- k_TT \mathbf{1}_{\mathbf{CT}}, \\ &\quad \mathbf{1}_{\mathbf{CT}} := \begin{cases} 1,&\text{for one day after chemotherapy application},\\ 0,& \text{else.} \end{cases} \end{aligned}$$

For the percentage of tumour cells *p*_*T*_ respectively effector cells *p*_*E*_ that were killed one day after chemotherapy, one obtains

$$\begin{aligned} p_T= 1-e^{-k_T}, \qquad p_E= 1-e^{-k_E}. \end{aligned}$$

The quantities *p*_*T*_ and *p*_*E*_ were used to illustrate the loss of cells due to single chemotherapy applications. Since the system is dominated by the direct cell kills shortly after chemotherapy application, the interaction between tumour and immune system can be neglected for estimating *p*_*T*_ and *p*_*E*_.

The right-hand side of model equations is Lipschitz continuous only for *T* bounded away from zero. On the other hand, we are interested in solutions only for *T*≥1. If *T* becomes smaller than 1, we consider the tumour as extinct. Application of chemotherapy introduces jump discontinuities at discrete time points. But Lipschitz continuity is guaranteed at the time intervals between applications. This implies that the overall solution is piecewise smooth.

#### The Principle of Dose Intensification

We need to determine the strength of dose intensified therapies in relation to a standard therapy. In order to keep the model simple, we make the following assumptions: CHOP is set as standard therapy. The chemosensitivity *k*_*T*_ and effector toxicity *k*_*E*_ of any other therapy are determined by a non-linear dose-toxicity-relation:

3 $$\begin{aligned} \begin{aligned}[c] & k_T^{\text{Intensified CHOP}} = k_T^{\text{CHOP}} \cdot D^{e_{T}}, \\ & k_E^{\text{Intensified CHOP}} = k_E^{\text{CHOP}}\cdot D^{e_{E}}. \end{aligned} \end{aligned}$$

*D* denotes the relative total dose standardised to CHOP. The total dose of a chemotherapy regimen is calculated as a weighted sum of the single drug doses employed. In Hasenclever et al. (2001), we described a method to estimate relative weights of cytotoxic agents based on a meta-regression analysis of chemotherapy comparing randomised clinical trials. Using this approach, we obtain *D*=1.34 for CHOEP-therapy. The parameters *e*_*T*_ and *e*_*E*_ describe the strength of toxicity increase by intensified doses for tumour cells and effector cells respectively. Values of *e*_*T*_ and *e*_*E*_ unequal to 1 express a non-linear dose-toxicity-curve as observed for haematotoxicity (Scholz et al. 2005).

### Parameters for Human Diffuse Large B-Cell Lymphoma

We aim to parameterise our model for humans. Since direct measurements of parameters are hardly available, it is necessary to determine parameters using biological knowledge and heuristic “up-scaling” of parameters identified for mice. This will require some simplifications and assumptions. Only a few parameters were later determined by fitting model predictions to clinical data.

Assuming that the biological mechanisms of the immune system do not differ substantially between mice and humans, we adopt the values from Kuznetsov et al. (1994) for the tumour-induced inactivation rate of effector cells *μ*_mouse_=*μ*_human_=3.422⋅10^−10^ day^−1^ cells^−1^ and the effector-induced elimination rate of tumour cells *ν*_mouse_=*ν*_human_=1.101⋅10^−7^ day^−1^ cells^−1^. We assess the remaining parameters either by reasonable transformation of the values from Kuznetsov et al. (1994) or by taking values from other references.

The effector cell inactivation rate is reported as *δ*_mouse_=0.3743 day^−1^ in Kuznetsov et al. (1994) which corresponds to a half life of $\frac{\ln(2)}{\delta_{\text{mouse}}} = 16\ \mbox{days}$. The half life of different human CD8+ cell populations is in the order of 100 days (Ladell et al. 2008). Hence, we set $\delta _{\text{human}}= \frac{\ln(2)}{100} \approx0.007\ \mbox{day}^{-1}$.

The production of effector cells *σ* is obtained by *σ*=*E*_equ human_⋅*δ* where *E*_equ human_ is the number of specific effector cells for humans in equilibrium, i.e. absence of tumour. From Alanio et al. (2010) and Coulie et al. (2002), we get an estimate of *E*_equ human_≈2⋅10^5^ cells. Hence, *σ*=*E*_equ human_⋅*δ*=2⋅10^5^⋅0.007=1400 cells⋅ day^−1^. We obtain similar values by another reasoning: The human body volume is estimated as around 1000 times the murine volume, and thereby *E*_equ human_=*E*_equ mouse_⋅1000 cells. With a murine tumour-free equilibrium state of *E*_equi mouse_≈200 cells (Blattman et al. 2002; Kedzierska et al. 2006; Moon et al. 2007), we finally obtain *E*_equ human_=200⋅1000=2⋅10^5^ cells. Note that *E*_equ mouse_ is markedly different from that originally assumed by Kuznetsov.

Interestingly, the ratio of the number of cells per clone to the number of clones specific for one antigen is different for mice and humans. It is 10/20 for mice and 5/40000 for humans (Alanio et al. 2010; Blattman et al. 2002). Thus, mice clones appear to have a lower specificity regarding antigens compared to humans. Hence, we assume that the increase of effector cells due to stimulation by tumour cells is more rapid in humans than in mice and that the saturation limit of effector cell turnover is reached earlier, i.e. *η*_human_=*η*_mouse_⋅0.01=2⋅10^5^ cells.

Some parameters are considered to be heterogeneous depending on the medical conditions of the patient or the dose intensity of the therapy. We only assess ranges for these parameters and fit distributions of them based on clinical data later (Sect. 3). Parameters assumed to be heterogeneous are the tumour growth rate *α*, the immunogenicity *ρ*, the tumour size at diagnosis *T*_diag_ and the effect of therapy on tumour cells *k*_*T*_ as well as on effector cells *k*_*E*_. Later, this heterogeneity reflects the patient heterogeneity with respect to therapy outcome.

The tumour doubling time for malignant lymphoma is reported as around 29 days in Tubiana (1989). We fix an interval between 1.4 and 70 days for DLBCL corresponding to a tumour growth rate *α* between 0.01 and 0.5 day^−1^ (*α*=ln(2)/(tumour doubling time in days)). The immunogenicity *ρ* is assumed as 0.1245 day^−1^ in Kuznetsov et al. (1994). We choose *ρ*∈[0.01,0.21] to cover a large range of possible parameters. We choose an elimination rate *p*_*T*_ between 0.01 and 0.9999 per dose application to describe the observed spectrum of highly sensitive tumours as well as therapy-resistant tumours: *p*_*T*_∈[0.01,0.9999]⇔*k*_*T*_∈[0.01,9.21]. The toxicity of effector cells *k*_*E*_ is assumed to be lower as the chemosensitivity *k*_*T*_, so we assume *p*_*E*_∈[0.01,0.80]⇔*k*_*E*_∈[0.01,1.6].

Furthermore, we assume heterogeneity of the tumour size at diagnosis *T*_diag_ as we want to model different stages of disease. We get an idea about the cell counts in an average large tumour by roughly estimating the number of tumour cells of a 200 ml sized tumour. The diameter of lymphocytes is reported in Abbas and Lichtman (2003) as about 10 μm 



We thus suppose that *T*_diag_ is in the order of 10^11^ cells and varies between 10^10^ and 10^13^ cells. The same scale is valid for relapse sizes. However, relapse size can be assumed to have smaller variances due to closer surveillance after therapy. For simplicity, we set a fixed tumour size of 10^11^ cells for relapse.

Table 1 presents a summary of the values and meanings of all model parameters and quantities.

### Qualitative Behaviour of the Model

We analyse the qualitative behaviour of the system without chemotherapy for the human parameters chosen in Table 1, i.e. we refer to parameters *σ* to *c*. Parameters *T*_diag_ to *k*_*E*_ refer to chemotherapy modelling only considered in Sect. 2.2. We analyse the steady states of the model described by Eq. (2). The steady states are determined by the nullclines *dE*/*dt*=0,*dT*/*dt*=0. Just like in model (1) there is a steady state at *T*=0, $E= \frac{\sigma }{\delta}$, which can be described as the tumour-free equilibrium state of effector cells. This is also a special case as the first equation of (2) is not differentiable in *T*=0. It is not possible to examine the stability of this steady state directly by determining the eigenvalues for the linearised system. However, we can deduce stability asymptotically. A steady state is stable if the Jacobian matrix **J** of the linearised system has a negative trace and a positive determinant. The sign of disc(**J**):=trace(**J**)^2^−4⋅det(**J**) determines whether the point is a node or a spiral. The Jacobian matrix for the linearised system (2) reads

$$\begin{aligned} \mathbf{J}(E,T) = \begin{pmatrix} a_{11} & a_{12}\\ a_{21} & a_{22} \end{pmatrix} = \begin{pmatrix} \frac{\rho T^c}{\eta+T^c} - \mu T^c-\delta& (\frac{\rho\eta }{(\eta+T^c)^2}-\mu)cET^{c-1}\\ -\eta T^c& \alpha-\nu cET^{c-1} \end{pmatrix} . \end{aligned}$$

For *T*→0 and *ρ*>*ημ*, it follows

$$\begin{aligned} a_{11} \rightarrow -\delta, \qquad a_{12} \rightarrow\infty, \qquad a_{21} \rightarrow0,\qquad a_{22} \rightarrow-\infty. \end{aligned}$$

With

$$\begin{aligned} {\mathrm{trace}}(\mathbf{J}) = a_{11} + a_{22} \rightarrow- \infty ,\quad \text{and}\quad {\mathrm{det}}(\mathbf{J}) = a_{11}a_{22}-a_{12}a_{21} \rightarrow\infty, \end{aligned}$$

we conclude that the steady state is stable and no saddle node. It holds that trace(**J**)=−*νcET* ^*c*−1^+*o*(*T* ^*c*−1^) and det(**J**)=*δνcET* ^*c*−1^−*o*(*T* ^*c*−1^) where *o* is the Landau order symbol. This leads to

$$\begin{aligned} {\mathrm{disc}}(\mathbf{J}) = \bigl(-\mathrm{trace}(\mathbf {J}) \bigr)^2-4\cdot{\mathrm{det}}(\mathbf{J}) = (\nu cE)^2T^{2c-2} - o\bigl(T^{2c-2}\bigr) > 0. \end{aligned}$$

It follows that this steady state is a stable node. Note that the tumour-free equilibrium state is always stable here, while in Kuznetsov et al. (1994) the stability of this fixed point depends on the parameters.

Furthermore, we obtain the nullcline of *T* from the second equation of the system (2)

4 $$ E= \frac{\alpha}{\nu} T^{1-c} $$

and therefore

5 $$ \log(E) = \log(\alpha/\nu) + (1-c) \log(T). $$

All remaining fixed points are located on a straight line with slope 1−*c* on the logarithmic scale which intersects the log(*E*)-axis at log(*α*/*ν*). An increase of *α* shifts this straight line in log(*E*)-direction.

The nullcline of *E* is

6 $$ E= \frac{\sigma(T^c+\eta)}{\mu T^{2c}+(\mu\eta+\delta-\rho )T^c+\delta\eta}. $$

**Fig. 2:**
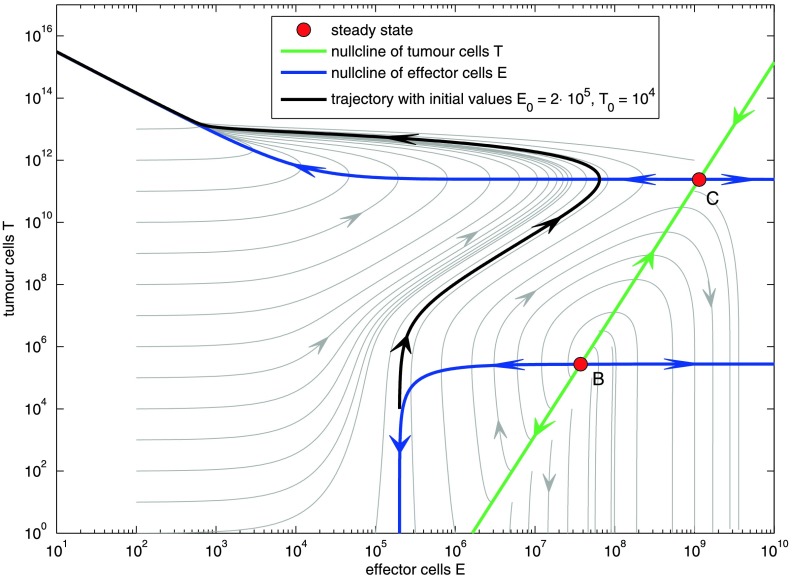
Phase diagram on the logarithmic scale for tumour growth rate *α*=0.18, immunogenicity *ρ*=0.1245 with nullclines and steady states. Steady state A is not shown. Also not shown is the tumour-free fixed point at *T*=0 and *E*=*σ*/*δ*=2⋅10^5^. Except for the latter one, all steady states are unstable

The curves of the nullclines are shown in Fig. 2.

Substituting (4) into the first equation of (2) yields the fixed-point equation for *T*:

7 $$ 0 = C_0 + C_1 T^{c} + C_2 T+ C_3 T^{(1+c)} + C_4 T^{(1-c)}, $$

where

$$\begin{aligned} &C_0 = \sigma\eta, \qquad C_1 = \sigma, \qquad C_2 = \frac{\alpha }{\nu} (\rho- \mu\eta- \delta), \\ & C_3 = - \frac{\alpha}{\nu }\mu, \qquad C_4 = - \frac{\alpha}{\nu}\delta \eta. \end{aligned}$$

Substitution of $Z = T^{\frac{1}{4}}$, for *c*=0.75 we obtain a polynomial of degree 7:

$$\begin{aligned} 0 = C_3 Z^7 + C_2 Z^4 + C_1 Z^3 + C_4 Z+ C_0. \end{aligned}$$

By applying Descartes’ rule of signs (Anderson et al. 1998), we are able to make statements regarding the number of positive roots. With

$$\begin{aligned} &\operatorname{sgn}(C_0) = 1,\qquad \operatorname{sgn}(C_1) = 1,\qquad \operatorname{sgn}(C_2) = \pm1,\\ & \operatorname{sgn}(C_3) = -1,\qquad \operatorname{sgn}(C_4) = -1, \end{aligned}$$

it follows that there are three changes of signs among the coefficients. This implies the existence of one or three positive roots. However, it is not possible to find an analytical solution of the real roots for general parameter values tumour growth rate *α* and immunogenicity *ρ*. Steady states were determined numerically using the statistical software package R (www.r-project.org, function “uniroot” in package “stats”). The function is based on the algorithm given in Brent (1973).

To illustrate model behaviour, we chose *α*=0.18 and *ρ*=0.1245 according to Kuznetsov et al. (1994). The qualitative behaviour of the model is the same for the range of possible values for *α* and *ρ* (not shown). Precise information about the number of distinct positive roots is given by Sturm’s method (Stoer 1989). There are four sign changes of the Sturm sequence for *Z*=0 and one sign change for *Z*→∞. It follows that there are three distinct real roots in [0,∞).

We get three numerical solutions for the steady states of Eq. (7) with *α*=0.18,*ρ*=0.1245:

8 $$\begin{aligned} &\mathrm{A} := (E_1,T_1) = \bigl(2 \cdot10^5,2 \cdot10^{-4}\bigr), \end{aligned}$$

9 $$\begin{aligned} &\mathrm{B} := (E_2,T_2) = \bigl(3.74 \cdot10^7, 2.74 \cdot10^5\bigr), \end{aligned}$$

10 $$\begin{aligned} &\mathrm{C} := (E_3,T_3) = \bigl(1.14 \cdot10^9, 2.4 \cdot10^{11}\bigr). \end{aligned}$$

The nature of the equilibria can be determined by linearisation of the system. The steady states are characterised as follows:

- A: saddle node,   B: unstable node or focus,   C: saddle node.

The system exhibits three steady states. Table 2 lists all steady states A, B, and C and corresponding ranges of E and T. Linearisation shows that the first steady state and the third steady state are always a saddle node and the second steady state is either an unstable node or an unstable focus.

**Table 2:** Numerical fix point analysis for *α*∈[0.01,0.5],*ρ*∈[0.01,0.21]. eig(**J**) denotes the eigenvalues of the Jacobian matrix, sgn(eig(**J**)) denotes the sign of the real parts of the eigenvalues

FP	min(*E*)	max(*E*)	min(*T*)	max(*T*)	eig(**J**)	sgn(eig(**J**))	class
A	2.00⋅10^5^	2.00⋅10^5^	3.78⋅10^−6^	2.37⋅10^1^	real	alternating	saddle node
B	1.66⋅10^6^	3.63⋅10^8^	1.11⋅10^5^	4.09⋅10^7^	real & complex	positive	unstable node/focus
C	1.84⋅10^7^	3.82⋅10^9^	1.62⋅10^9^	4.98⋅10^11^	real	alternating	saddle node

The phase diagram of ODE (2) with the parameter values from Table 1 is illustrated in Fig. 2. It can be divided into two domains.

**Tumour overgrows immune system**: An initially small tumour population stimulates the immune system by increasing the effector cell population. By reaching a size large enough to weaken the immune system, the tumour depletes the effector cell population to 0.
**Tumour eventually becomes extinct**: There are two distinct dynamics here: Either a small tumour population becomes extinct immediately. Or, after an initial growth of both tumour cells and effector cells, the tumour is eliminated by the effector cells.

We can study the characteristics of the system by examining the nullclines (Eqs. (4) and (6)) in Fig. 2:

- **T-nullcline** (green line in Fig. 2): the tumour size increases for effector cell populations lying on the left of this straight line and decreases for effector cell populations lying on the right side of it.
- **E-nullcline** (blue curve in Fig. 2): the effector cell population increases in between the two blue curves. It decreases above the upper curve and below the lower curve.

**Fig. 3:**
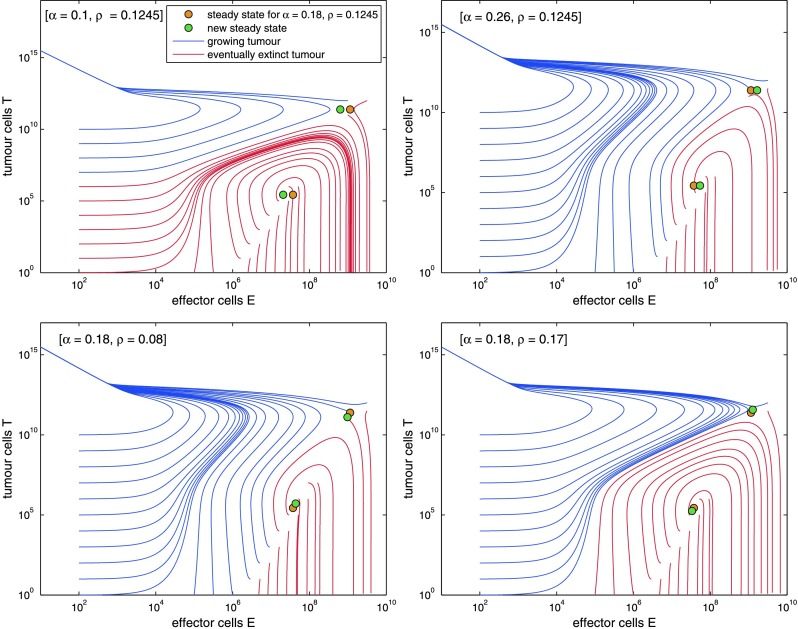
We study dependence of qualitative model behaviour on settings of the parameters tumour growth rate *α* and immunogenicity *ρ*. Settings used by Kuznetsov et al. served as a basis (*α*=0.18, *ρ*=0.1245). Phase diagrams for larger or smaller values are presented. Large tumour growth rate *α* as well as small immunogenicity *ρ* result in smaller areas of attraction for tumour elimination. Large *ρ* results in a steeper peak of *E*

Figure 3 illustrates the influence of the tumour growth rate *α* and the immunogenicity *ρ* on the system behaviour. Decreasing *α* respectively increasing *ρ* enlarges the domain where the tumour eventually is eliminated. Additionally the quantity of immunogenicity *ρ* determines the steepness of the effector cell curve: a higher *ρ* implies a steeper peak. Note that similar phase diagrams can be obtained by either increasing tumour growth velocity *α* or decreasing immunogenicity *ρ*. In the first case, the immune system has not enough time to react against the fast growing tumour, while in the second case the immune system is insufficiently stimulated.

## Linking the Model to Clinical Data

In this section, we describe how the model is linked to clinical data in order to estimate unknown parameters of the model (see Sect. 2.3). The biological heterogeneity of patient populations is described by a joint distribution of five relevant model parameters: tumour growth velocity *α*, immunogenicity of the tumour *ρ*, size of the tumour *T*_diag_, and chemosensitivity of tumour *k*_*T*_ as well as effector cells *k*_*E*_. We aim to estimate this multi-dimensional distribution from survival data of randomised chemotherapy trials.

For this purpose, the ODE system is solved on a multi-dimensional hyper-cube parameter grid. Each grid point represents a constellation of the model parameters that were assumed to be heterogeneous in a patient population. For each grid point we determine whether the tumour growth reaches diagnosis volume and whether the tumour would be cured by the chemotherapy given. We also calculate the time of relapse if applicable.

Distributions on the parameter grid will be chosen by the maximum entropy condition with a few moment constraints on means or variances (Jaynes 1957). The constraints comprise expectation and variance of immunogenicity *ρ*, tumour volume at diagnosis *T*_diag_ and chemosensitivity *k*_*T*_ as well as expectation of tumour growth rate *α* (see Table 4 below). A specific distribution of parameters implies a survival curve. An evolutionary algorithm is then used to fit the model prediction to data.

In summary, we parameterised the model by the following steps:

1. Simulation of all therapies on the discrete grid of parameter combinations.
2. Specification of moment constrains of distributions on the grid.
3. Determination of Maximum-Entropy probability distribution on the parameter grid fulfilling the moment constrains.
4. Determination of time-to-progression curves for specified therapies given the probability distribution on the parameter grid.
5. Optimisation of moment constrains to minimise differences between study data and survival curves predicted by the model.

### DLBCL Chemotherapy Studies Used for Model Calibration

Table 3 lists the studies used for model calibration with corresponding regimes, event-free survival rates (EFS), and some prognostic factors of the patient populations. EFS was defined as the time from the beginning of therapy to either disease progression, relapse, or death. The long-term standard chemotherapy for diffuse large B-cell lymphoma was 6xCHOP-21 which consists of six times cyclophosphamide, doxorubicin, vincristine, and prednisone, given every 3 weeks (Fisher et al. 1993). In the NHL-B2 study (Pfreundschuh et al. 2004a,b), the effectiveness of two-weekly CHOP and intensified CHOP with etoposide relative to standard CHOP was investigated. The patient population comprised 689 patients aging between 61 and 75. It is observed in NHL-B2 that both, addition of etoposide and time intensification results in a better outcome, but that these factors are not additive. Moreover, CHOEP-14 is worse than CHOP-14, an effect considered as paradoxic here (see Fig. 1).

**Table 3:** Clinical study overview

Study Name	Age	Study arms and 3-year EFS rates			
NHL-B2	61–75	6xCHOP-21 41.3	6xCHOP-14 54.2	6xCHOEP-21 45.5	6xCHOEP-14 46.0
RICOVER-60	61–80	6xCHOP-14 47.2	8xCHOP-14 53.0		

**Fig. 4:**
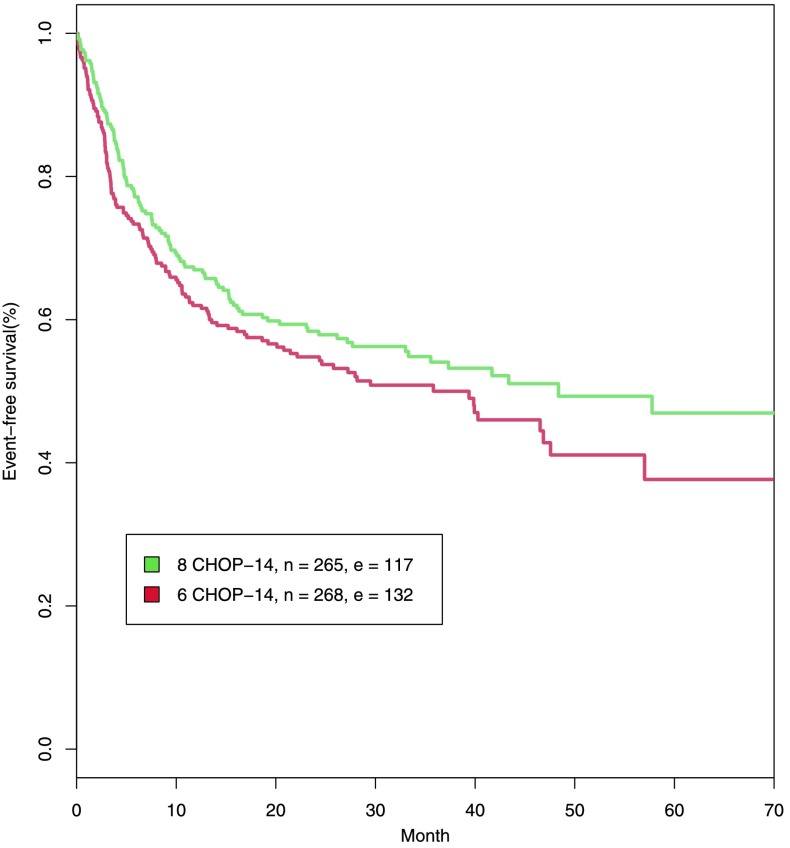
Event-free survival in months for patients between 61 and 75 years from RICOVER-60. *n* and *e* indicate the number of patients and the number of events

The RICOVER-60 trial was designed to compare six versus eight cycles of bi-weekly CHOP-14 with or without Rituximab (Pfreundschuh et al. 2008). 1,222 patients between 61 and 80 years were randomised. Since the effect of Rituximab is not modelled here, we only consider the six and eight cycles CHOP-14 EFS-curves of the treatment arms without Rituximab. Eight cycles of CHOP-14 result in a better outcome than six cycles of CHOP-14 (see Fig. 4).

### Adjusting Different Studies with Similar Patients

Patient characteristics are rather similar for RICOVER-60 and NHL-B2 after restricting the age range to 61–75 years. Thus, we assume the same parameter set for these two different studies. However, the EFS curves of six cycles of CHOP-14 differ slightly between the studies, probably due to differences in recruitment practice. To avoid estimation of a completely new parameter set for RICOVER-60, we adjust for this (random) study effect by introducing a parameter *lhr* that adapts the survival rates of NHL-B2 to RICOVER-60 assuming proportional hazard. This parameter needs to be fitted, also.

### Modelling Patient-, Therapy-, and Study-Specific Heterogeneity of Parameters

Some parameters are considered as patient-, therapy-, or study-specific (see Table 4), defined as follows:

**Patient-specific:** These parameters differ between patients. A population distribution is assumed for these kind of parameters.
**Therapy-specific:** The parameter value depends on the applied chemotherapeutic drugs and drug doses.
**Study-specific:** The parameter value varies for different studies with similar patient populations.

**Table 4:** Overview of fitted parameters and distributions for NHL-B2 and RICOVER-60. The heterogeneity of the parameters is either on a patient-level, therapy-level, or study-level (see text). The standard deviation for tumour growth rate *α* distribution is not explicitly specified but is a result of the maximum entropy condition

Fitted parameter distribution	Heterogeneity	Distribution parameters prescribed (constraints for max. ent.)	Fitted value	5/95 %-Percentile	Remark
Tumour growth rate *α*	patient specific	expectation	0.118	[0.039,0.216]	fitted
		standard deviation	0.054		result of max. entr. cond.
Immunogenicity *ρ*	patient specific	expectation	0.078	[0.022,0.145]	fitted
		standard deviation	0.038		fitted
log10(tumour volume *T*_diag_)	patient specific	expectation	11.31	[10.5,12]	fitted
		standard deviation	0.47		est. from data
Chemosensitivity *k*_*T*_	patient & therapy specific	expectation	2.36	[1.86,3.0]	fitted
		standard deviation	0.31		fitted
Effector tox *k*_*E*_	therapy specific	single parameter	0.48		fitted
dose exponent tum. cells *e*_*T*_	therapy specific	single parameter	0.80		fitted
Dose exponent eff. cells *e*_*E*_	therapy specific	single parameter	2.44		fitted
Prop. hazard factor *lhr*	study specific	single parameter	0.164		fitted

Tumour growth rate *α*, immunogenicity *ρ* and tumour volume at diagnosis *T*_diag_ are patient-specific while chemosensitivity *k*_*T*_ is patient- and therapy-specific. Chemotherapy toxicity to immune cells *k*_*E*_ is considered to be therapy-specific, but not patient-specific. Finally, the correction factor of hazard rates *lhr* between studies of the same population is study-specific. Plausible ranges of parameters were determined in Sect. 2.3. The standard deviation for tumour size at diagnose *T*_diag_ calculates to $\operatorname{SD}(T_{\text{diag}}) = 0.042\cdot \operatorname{E}(T_{\text{diag}})$. This is based on a variation coefficient of 0.042 retrieved from log tumour volume data of the EuroNet-PHL-C1 study (personal communication D. Hasenclever).

#### Simulation of Chemotherapy

We simulate the application of chemotherapy for an arbitrary parameter combination in two steps: At first we determine if these parameters are *admissible*, i.e. we check whether a tumour grows to the diagnosable size of *T*_diag_ for a specific parameter combination (*α*,*ρ*,*T*_diag_) or whether it is suppressed by the immune system before it is clinically apparent. The parameter distribution to be estimated is restricted to this admissible set. We start with an initial condition of *T*=10^4^ and *E*=*σ*/*δ*=2⋅10^5^, which describes the immune equilibrium state. The exact choice of *T*(0) is unimportant for model behaviour as long as one starts with a sufficiently small tumour, i.e. as long as the loss of effector cells due to presence of tumour represented by parameter *μ* can be neglected compared to the natural degradation of effector cells represented by parameter *δ*. In case of a growing tumour, we determine the effector cell population *E*_diag_ if *T*=*T*_diag_ is reached for the first time. In the second step, we simulate the therapy by starting at the initial condition (*T*_diag_,*E*_diag_) for each parameter combination (*α*,*ρ*,*T*_diag_,*k*_*T*_,*k*_*E*_). Therapy regimes vary in the number of cycles (6 or 8), time between dose application (e.g. 2 or 3 weeks), and chemotherapy dose expressed by the dose-dependence of *k*_*T*_ and *k*_*E*_ (see Eq. (3)). For each therapy, we determine if and when relapse occurs. Thus, we obtain therapy results in form of time to event data.

### Parametrisation of the Model

#### Discretisation of the Parameter Space

We discretise the ranges of the parameters by introducing grid points. Therapy simulation is performed on the grid for each parameter combination (*α*,*ρ*,*T*_diag_, *k*_*T*_, *k*_*E*_). Altogether we consider 51(*α*)⋅35(*ρ*)⋅7(*T*_diag_)⋅41(*k*_*T*_)⋅31(*k*_*E*_) grid points in the five-dimensional space. For instance, we used the seven grid points {10^10+*i*/2^,*i*=0,…,6} as possible tumour sizes at diagnose *T*_diag_. Density of the grid is chosen in dependence on the corresponding parameter sensitivity of the fitness function. If small changes of a parameter results in large differences of the fitness function, the grid was chosen finer to achieve a better approximation.

We implemented the simulation algorithm in MATLAB. The exact simulation settings are available on request.

#### Calculating the Maximum Entropy Probability Distribution

A four-dimensional distribution is assumed for the patient-specific tumour growth rate *α*, the immunogenicity *ρ*, the tumour size at diagnose *T*_diag_ and the chemosensitivity *k*_*T*_. Thus, we assign a probability distribution to the four-dimensional simulation grid since *k*_*E*_ only depends on therapy.

We use the maximum entropy principle (Jaynes 1957; Kullback 1959) to determine probability distributions on the four-dimensional parameter grid. We specify certain moments (means, variances, see Table 4) and determine the unique probability distribution, which fulfils these constraints and has maximum entropy. Given our parameter ranges and data, the maximum entropy condition implies unimodal distributions on the parameter grid. This appears to be biologically plausible because sub-groups of patients with largely different biology are not described so far. We used the package minxent of R (Kapur and Kesavan 1992) to solve this variational problem.

#### Computation of the Fitness Value

The fitness value measures the agreement of model and data as a function of the corresponding parameter distribution. To determine the fitness value, we use the time to event data and the probability of each parameter combination for any therapy to compute event-free survival rates. We obtain an EFS curve for each therapy by linear interpolation of the EFS-rates. We compare it to observed Kaplan–Meier estimates by computing the Euclidean distance for time points with a distance of 25 days between day 200 and 725. Time points earlier than 6 months after start of therapy were not considered due to reporting bias and toxic side effects during therapy. Time points greater than 25 months after start of therapy are influenced by late relapses not covered by our model. The fitness value is the sum of all of the above mentioned distances of the therapies considered.

#### Optimisation by Evolutionary Principle

An evolutionary algorithm is used to fit the model. The idea of evolutionary algorithms is based on the generation of new parameter sets by random variation of an existing parameter set with adapted step size and selection of a set with better fitness. Here, we estimate a parameter combination, which consists of moment constraints such as expectations and variances of the model parameters; see Table 4. A (1+5)-ES (Evolutionary Strategy) is used which means that 5 mutant parameter combinations are generated, which compete with their parent. For details about the concept of evolutionary algorithm, see e.g. Ashlock (2006), Schwefel (1984). The algorithm was implemented in R.

## Results

### Qualitative and Quantitative Model Results

We estimated the parameters of Table 4 with the method presented in Sect. 3. We assumed the same parameter set for the patients of NHL-B2 and RICOVER-60 except for the log hazard factor used to adjust the differing survival curves.

**Fig. 5:**
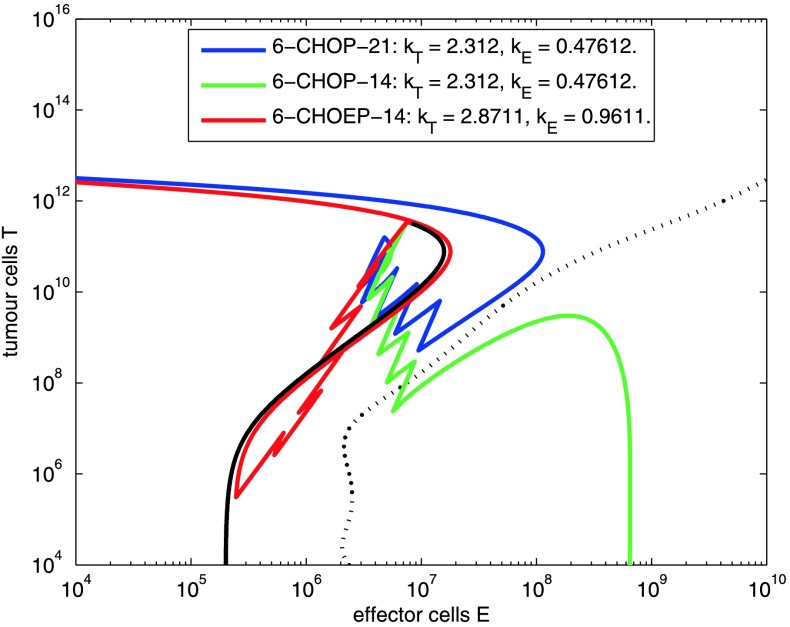
Parameter setting where moderate intensification of CHOP-21 (CHOP-14) leads to cure, but stronger intensification results in relapse (CHOEP-14). The *black trajectory* describes tumour growth prior to therapy. *Coloured trajectories* correspond to different therapy regimens

The model gives a possible explanation of therapy effects observed in NHL-B2 as illustrated in Fig. 5. The course of 6 cycles CHOP-14/21 and CHOEP-14 is shown in the phase space of effector and tumour cells for a typical parameter combination. Here, standard 6 cycles CHOP-21 is insufficient to eliminate the tumour. Time reduction between dose application pushes the course below the separatrix and results in complete elimination of tumour cells. Both time and dose intensification results in an even smaller number of tumour and effector cells. However, it is above the separatrix, and thus finally results in relapse.

**Fig. 6:**
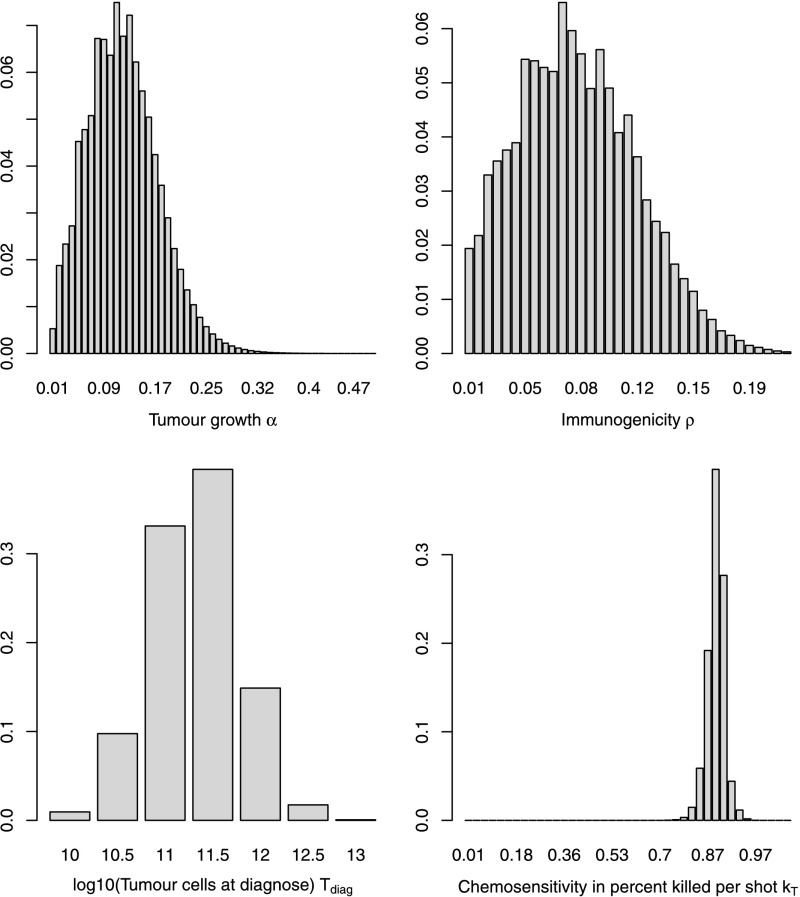
Marginal distribution densities of tumour growth rate *α* (*top left*), immunogenicity *ρ* (*top right*), tumour volume *T*_diag_ (*bottom left*), and chemosensitivity *k*_*T*_ (*bottom right*)

Marginal distributions of the parameters are shown in Fig. 6. The asymmetric shape of the distribution is a result of restricting parameters to admissible parameter sets.

Parameters can be read from Table 5: The expected tumour growth rate *α* was estimated to be 0.118, which corresponds to a potential tumour doubling time of 5.88 days. The standard deviation is 0.054. 90 % of values are between 0.039 (doubling time 17.6 days) and 0.216 (doubling time 3.2 days). The immunogenicity *ρ* has an expected value of 0.078. The expected tumour load *T*_diag_ is 11.31 on log10-scale. The expected chemosensitivity *k*_*T*_ is 2.36, which implies a tumour kill of 90.6 % per shot. 90 % of values are between 1.86 (84.3 % kill per shot) and 3.00 (95.0 % per shot). The effector toxicity *k*_*E*_ is 0.48 resulting in a kill of 37.9 % effector cells per shot. These values indicate that the grid is chosen adequately as the values are not located on the edge of the grid. The toxicity exponent for intensified therapies (see Eq. (3)) is 0.80 for tumour cells and 2.44 for effector cells leading to an expected cell kill of 95.0 %, respectively, and 62.2 % for intensified Etoposide therapies. Note that it is much higher for effector cells than for tumour cells. Finally, the proportional hazard factor *lhr* to adjust the RICOVER-60 survival data to NHL-B2 is 0.164.

**Table 5:** We calculated univariate confidence intervals for parameter estimates by allowing 10 % deterioration of fitness value

Parameter	Interval	In percent
EXP(log10(tumour volume *T*_diag_))	[*T*_diag_−0.028;*T*_diag_+0.023]	[−0.3 %;+0.2 %]
EXP(immunogenicity *ρ*)	[*ρ*−5⋅10^−4^;*ρ*+6⋅10^−4^]	[−0.6 %;+0.8 %]
EXP(tumour growth *α*)	[*α*−9⋅10^−4^;*α*+7⋅10^−4^]	[−0.8 %;+0.7 %]
EXP(chemosensitivity *k*_*T*_)	[*k*_*T*_−0.022;*k*_*T*_+0.025]	[−0.9 %;+1.1 %]
effector toxicity *k*_*E*_	[*k*_*E*_−2⋅10^−4^;*k*_*E*_+0.013]	[−0.05 %;+2.7 %]
CHOEP exponent eff. tox *e*_*E*_	[*e*_*E*_−0.141;*e*_*E*_+0.114]	[−5.8 %;+4.7 %]
SD(immunogenicity *ρ*)	[sd(*ρ*)−0.003;sd(*ρ*)+0.003]	[−6.9 %;+7.1 %]
SD(log10(tumour volume) *T*_diag_)	[sd(*T*_diag_)−0.041;sd(*T*_diag_)+0.030]	[−8.8 %;+6.2 %]
CHOEP exponent chemosens *e*_*T*_	[*e*_*T*_−0.061;*e*_*T*_+0.078]	[−7.6 %;+9.8 %]
SD(chemosensitivity *k*_*T*_)	[sd(*k*_*T*_)−0.069;sd(*k*_*T*_)+0.048]	[−22.5 %;+15.6 %]
prop. hazard factor *lhr*	[*lhr*−0.048;*lhr*+0.040]	[−29.1 %;+24.1 %]

**Fig. 7:**
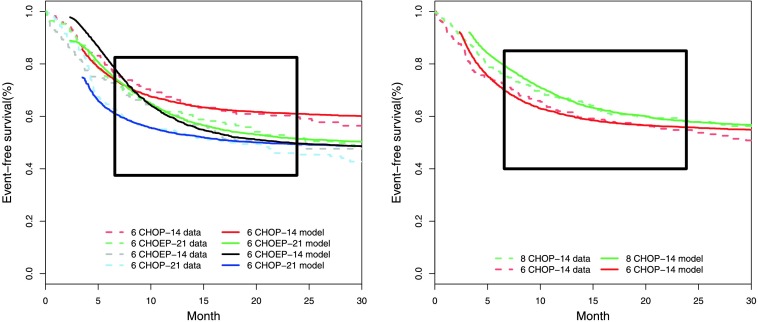
Comparison of model and data for NHL-B2 (*left*) and RICOVER-60 (*right*). Model is fitted within the range between day 200 and day 725

**Fig. 8:**
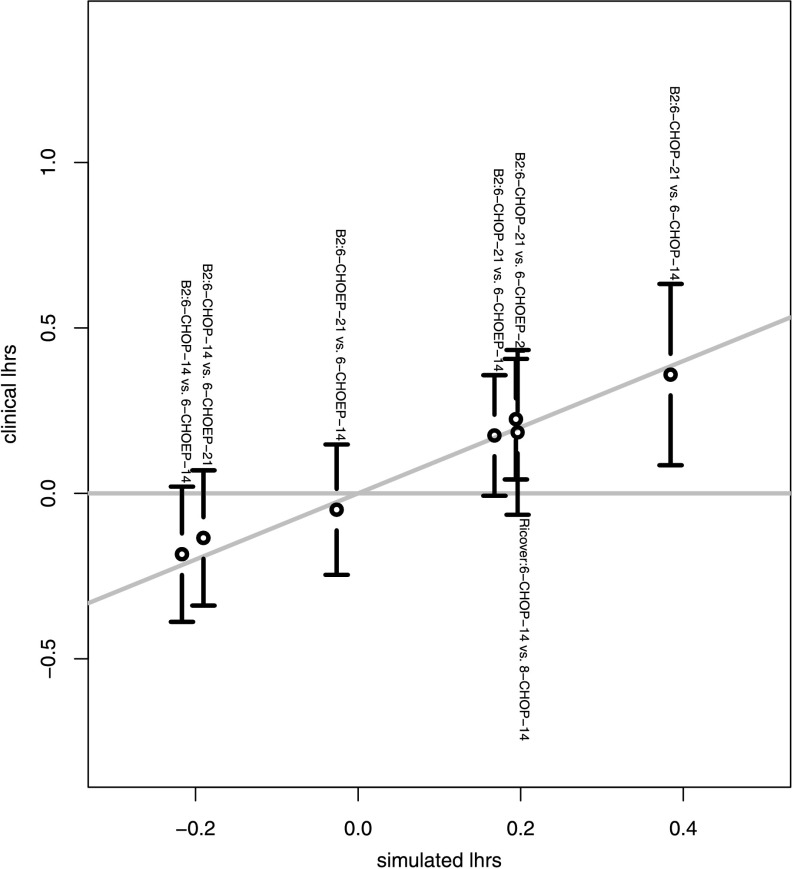
Pairwise log hazard ratios of the model vs. 95 %-confidence intervals of log hazard ratios of the data

Fitted survival curves are shown in Fig. 7. We also displayed the log hazard ratios of all combinations of treatment arms for each study against the log hazard ratios of the model-simulated survival curves (Fig. 8). We observed a good agreement of model and data within the time-frame of validity of the model. The model explains all therapy contrasts in the sense that the prediction is always in the confidence interval of the log hazard ratio of the data. In particular, the model reproduces paradoxic therapy effects: The outcome of CHOEP-14 is worse than that of CHOP-14 in NHL-B2.

**Fig. 9:**
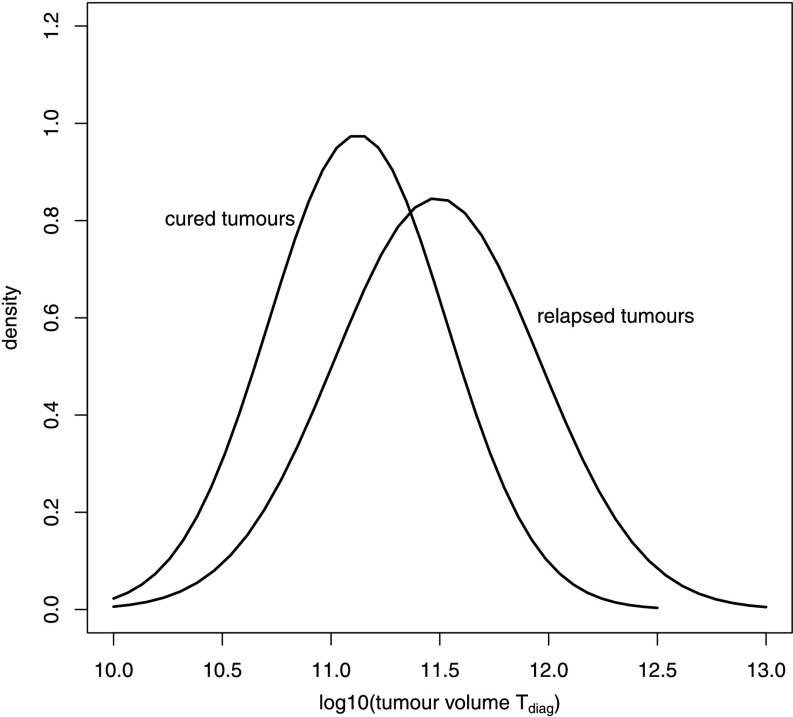
Tumour load (*T*_diag_) distributions of relapsed and cured tumours. A sample of *N*=10 000 was drawn from the fitted parameter distribution. Corresponding outcomes of six cycles CHOP-21 therapy were determined. A normal distribution was fitted to the initial tumour loads of cured and relapsed patients, respectively

It is common practice to distinguish between advanced and low states of the tumour. Figure 9 shows the distributions of tumour size of cured and relapsed patients populations. We observe as expected that relapsing tumours have higher average initial sizes than cured tumours (Fig. 9).

The sensitivity of each parameter is examined in Table 5. Parameters are sorted in descending order in dependence on sensitivity. We observe that parameters representing distribution means are more sensitive than parameters representing variances.

### Model Predictions

**Fig. 10:**
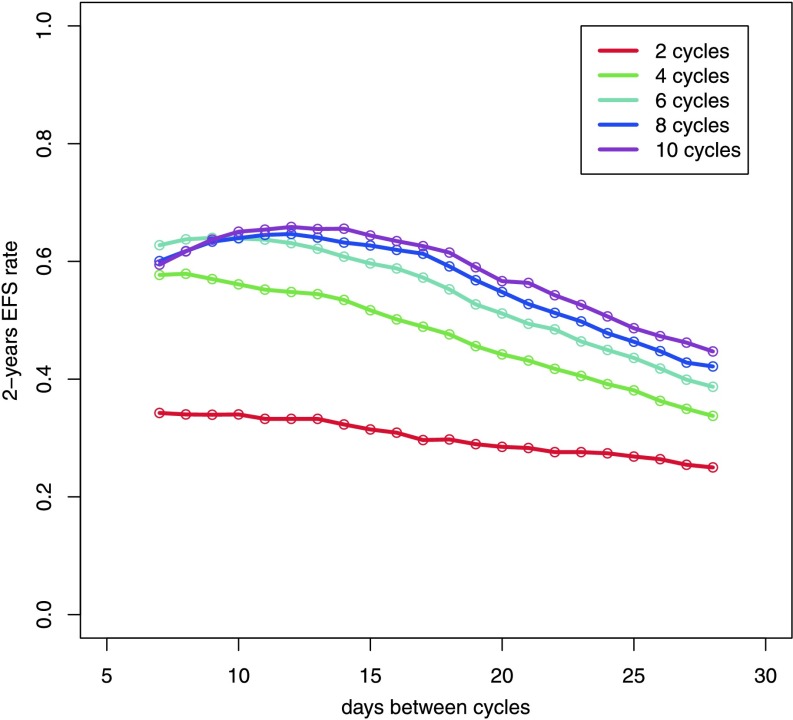
Prediction of 2-year EFS rates as a function of number of chemotherapy cycles and time between chemotherapy administration

There is ongoing research regarding required number of cycles and the effect of time intervals between them. By our model, we aim at contributing to this discussion by systematically simulating 2, 4, 6, 8, and 10 cycles of CHOP with varying cycle duration (7 to 28 days) and predicting corresponding estimates of 2-years survival (Fig. 10). It turns out that two cycles are clearly insufficient and four cycles are sub-optimal, also. The gain achieved by more than six cycles is relatively small. Compared to CHOP-14, we predict only limited improvement of therapy by further time intensifications. Two-year survival clearly drops if therapy intervals are greater than 21 days.

Although there is uncertainty in model parameters, we observed that 2-year survival rates are robust against changing model parameters. This is illustrated by the therapies 6xCHOP-14, 6xCHOP-12 and 4xCHOP-14 simulated for upper and lower confidence limits of parameter predictions (Table 6).

**Table 6:** Effect of parameter uncertainty on model predictions, we used upper and lower confidence bounds of parameters as presented in Table 5 and calculated maximal deviations from predicted 2-year-EFS

Study comparison	2-years-EFS	Min(2-years-EFS)	Max(2-years-EFS)
4-CHOP-14	0.53	0.52	0.54
6-CHOP-12	0.63	0.62	0.64
6-CHOP-14	0.61	0.60	0.62

## Discussion

Models of tumour growth and chemotherapy induced tumour cell kill are typically incompatible with the notion that more intense therapy can be less effective. The NHL-B trial provides such an example. Therefore, we investigated the idea that the immune system is important to control remaining tumour cells. Paradoxical treatment outcomes can occur when intensive chemotherapy shuts down the immune system in the critical time window at the end of therapy.

Figure 5 illustrates that the simple ODE-system described here features paradoxical treatment effects. In addition, we linked our ODE-model to clinical data by describing latent patient heterogeneity by a distribution of specific model parameters. This distribution can be estimated from clinical trial data using an innovative method. Our model can be made quantitatively consistent with progression-free survival curves (Fig. 7) and observed treatment effects (Fig. 8) from several trials. The respective parameter estimates appear plausible.

Our approach relies on a number of simplifications and has some limitations, which we discuss in the following:

### Alternative Models of Tumour and Immune Response

The model of tumour and immune system interaction in mice proposed by Kuznetsov et al. (1994) served as a backbone of our modelling. We use a modified and simplified version of it as a minimal model to address tumour growth and immune response. Numerous more complicated models were proposed. Gałach (2003) modified the model of Kuznetsov et al. (1994) by replacing the Michaelis–Menten form of the tumour induced immune stimulation term with a Lotka–Volterra term. An effect of time delay of the immune response was also introduced. Recently, the model was extended by Letellier et al. (2013). de Vladar and González (2004) assume a Gompertzian growth of the tumour population. The stimulation and loss of the immune system is modelled in a different way. However, model behaviour is similar to Kuznetsov’s model. Sotolongo-Costa et al. (2003) proposed a model of periodical immunotherapy with cytokines on the basis of the model of Kuznetsov.

de Pillis et al. proposed much more detailed models of immune response differentiating between Natural Killer cells (NK cells), CD8+ cytotoxic T-cells and other lymphocytes (de Pillis et al. 2005, 2006; de Pillis and Radunskaya 2000). This results in ODE models with several equations modelling the dynamics of tumour cells, NK cells, CD8+T cells, other lymphocytic cells, immunotherapy drug concentration and chemotherapy drug concentration. Another detailed model of immune response considering the interactions of cancer cells, NK-cells, lymphokine-activated killer cells, cytotoxic T-cells, helper T-cells and B-cells was presented by Szymańska (2003). Kirschner and Panetta (1998) modelled the dynamics between tumours cells, effector cells, and the cytokine interleukin-2 (IL-2), which serves as a modulator of the immune stimulus. Arciero et al. (2004) extended this model by considering the tumour escape effect induced by TGF-*β* and corresponding siRNA treatment.

Page and Uhr (2005) proposed different models of tumour dormancy by explicitly modelling the dormant cell population. They neglected the effect of antigen-specific T-cells, but modelled the immune response by an equation describing the effect of antibodies in murine BCL1 lymphoma. A complex model with 10 ODEs and 3 additional equations describing several players of the immune response is given by De Boer and Hogeweg (1986). This model also covers all phenomena from uncontrolled tumour growth to tumour regression due to immune response.

So far, none of the models are designed to explain data of patients under chemotherapy. Dynamics of tumour load are not assessable for humans. Therefore, patient survival data are the only source for parameterising models for the human situation. Since these data are much less informative, we intentionally chose the simplistic version of Kuznetsov et al. (1994) as a basis of our modelling. This model is characterised by a single surrogate effect of the immune system, which best fits to the CD8+ cytotoxic T-cell population (Kuznetsov et al. 1994).

### Modifications of the Kuznetsov Model

Instead of a logistic growth, we assumed an exponential growth term of the tumour since simulations of our model were usually stopped if a certain tumour amount is exceeded (relapse). At this volume, the growth is far below saturation, and thus, our simplification is reasonable (Norton and Simon 1979). We assumed a “fractal” tumour dimension by applying an exponent *c* to the tumour load for terms describing the interaction of tumour and immune system. This is based on the observation that lymphoma grow in several lesions rather than in one compact node. The constant *c* is in between 2/3 for single node tumours and 1 for disseminated disease. For simplicity, we assumed *c*=0.75.

The modified model has a different attractor landscape as compared to Kuznetsov et al. (1994) for relevant parameter settings. It turns out that there are only two attractors, namely the tumour grows to infinity while the immune system becomes extinct or the tumour is eliminated and the immune system reaches its steady state. The latter one has the drawback that small amounts of tumour cells are automatically eliminated under specific parameter constellations. Thus, the model does not explain the development of a tumour in an early phase. Our ODE representation is more suitable for describing the effects at larger cell numbers neglecting stochastic effects of small cell numbers. The latter one would require a completely different approach, i.e. by constructing agent based models. For the same reason, with our model we cannot explain late relapses (after more than 2 years), which are probably caused by awaking dormant tumour cells not considered here. The original Kuznetsov model comprise parameter constellations for which there is a stable steady state of small tumour amounts controlled by a stimulated immune system. A possibility to cover this behaviour in our model would be to increase the exponent *c* to 1 for small tumours in Eq. (2).

### Incorporating the Effects of Chemotherapy

Chemotherapy is introduced by a transient first-order loss of both tumour and immune cells for the duration of one day after chemotherapy application. The dependence of corresponding toxicity parameters on the drug or drug concentration was modelled by a simple power function of the effective dose of a drug introduced in Hasenclever et al. (2001). Alternatively, one has to model each single drug of a polychemotherapy system, which would increase the number of free parameters. Blood cell toxicity is also modelled by first-order loss and a power law dependence on drug concentration. Both assumptions proved adequate in models of haematopoiesis under chemotherapy (Scholz et al. 2005, 2006). We also considered alternative approaches to model the effect of chemotherapy on the immune system by assuming reductions of the production rate of effector cells *σ* or the rate of effector cell stimulation by the tumour *ρ*. However, in our hands, these scenarios do not result in paradoxic therapy effects (results not shown).

### Identification of Model Parameters

Since Kuznetsov’s model was parameterised for the murine system, it was necessary to re-parameterise the model for patients. We retrieved parameter estimates or parameter ranges from the literature or set parameters to biologically or clinically plausible values.

To model heterogeneity of patients, we assumed that the parameters tumour growth velocity *α*, immunogenicity of tumour *ρ*, chemosensitivity of tumour *k*_*T*_, and volume at diagnosis *T*_diag_ can vary within a certain parameter range. We estimated the distribution of these patient specific parameters by fitting the predictions of our model to clinical survival data. We developed an innovative algorithm to solve this task: At first, we simulated a discrete grid of parameter values. Then we determined the maximum entropy distribution parameterised by certain moment constrains on the grid, which induces a survival curve. The agreement of survival curve and clinical data is optimised by evolutionary strategies. Kirkby et al. (2007) used a stochastic approach to fit their tumour model on clinical survival data. This is performed by drawing parameter values from assumed distribution families, comparing corresponding model results with clinical survival data and optimising the agreement with respect to the parameters of the distribution family. This approach was not feasible for our model in view of the computational burden induced by solving the differential equation system multiple times.

Only a few parameters with large impact on survival curves were determined by fitting model predictions to clinical data, namely expectation and variance of immunogenicity *ρ*, chemosensitivity of tumour *k*_*T*_, tumour size at diagnosis *T*_diag_, expectation of tumour growth velocity *α*, and the fixed value of chemosensitivity of immune cells *k*_*E*_. In consequence, there is still considerable uncertainty regarding model parameters. We extensively analysed the sensitivity of model parameters when establishing the model. It turned out that baseline production of immune cells (*σ*) and Michaelis–Menten term (*η*) have a relatively small impact on model behaviour since they are only relevant for small tumour sizes. Similarly, for large tumour sizes, the second term of the first model equation is linear in *E* so that the fourth term (containing parameter *δ*) simply implies a shift of the distribution of immunogenicity (*ρ*). Rather than the single values, the quotient of the mutual destruction rates *μ* and *ν* is relevant. It has a larger impact on the quantitative model behaviour but the same results can be achieved by alternative distributions of the other model parameters (results not shown). Better data are required to remove the uncertainty of model parameters in the future. For example, time series data of different immune cell fractions and surrogate markers of tumour load would be helpful.

In general, we considered both means and standard deviations as moment constrains of our multidimensional parameter distribution. Our sensitivity analysis revealed that our mean estimates are substantially better determined than those of the standard deviations. We assumed no (prior) correlation between parameters. However, due to the restriction to admissible parameter sets (compare Sect. 3.3), large values of immunogenicity *ρ* imply large values of tumour growth velocity *α* (see Fig. 3). Thus, we decided to drop a moment constraint regarding variance of tumour growth velocity *α*. As a biological consequence of these considerations, we predict that quickly proliferating tumours are better detected by the immune system which can be explained by a generally higher metabolic activity of tumour cells resulting in higher production rates of cytokines.

### Parameter Estimates are Biologically Plausible

The mean tumour doubling time was estimated as about 6 days with 90 % of values between 3 and 18 days. It is well known that aggressive non-Hodgkin’s lymphoma are highly proliferating with a tumour doubling time between 24 hours and about 30 days in dependence on the histologic subtype (Frolund et al. 2011; Lang et al. 1980; Tubiana 1989). The standard deviation of tumour growth *α* 0.054 is rather large referred to our grid. This is also plausible as the patient collective of NHL-B2 is heterogeneous (lactate dehydrogenase levels (LDH) normal and greater than normal, complete range of the International Prognostic Index is covered). The tumour stimulation rate of effector cells *ρ*=0.078 is difficult to interpret as it comprises many aspects of the immune system. However, it is in the same order of magnitude compared to Kuznetsov’s estimate of 0.1245 for mice. The expected tumour volume at diagnose *T*_diag_ is 10^11.31^. This fits well to our understanding because in this magnitude the turnover of effector cells is also high, i.e. the tumour becomes symptomatic.

### Limitations in the Fit to Clinical Outcomes

Within the time-frame used for model fitting, parameter estimates resulted in a good agreement of model and survival data of the two randomised clinical trials NHL-B2 and RICOVER-60 comprising five different chemotherapy regimens (Pfreundschuh et al. 2004b, 2008). Predicted hazard ratios between therapy options were in agreement with the observed data, i.e. the paradoxic therapy effects are explained by the model. On the other hand, we have to acknowledge that our model is invalid during therapy possibly due to reporting bias of progresses and toxic side effects under therapy. Additionally, our model cannot explain late relapses, which probably would require introducing elements of stochasticity to model dynamics of residual disease. Note that our equations are only valid for larger cell populations.

### Model Prediction and Outlook

To demonstrate practical use of our model, we performed simulations of modified CHOP regimens by varying therapy intervals and number of cycles. We predicted for example that six cycles of CHOP-14 are necessary for reasonable cure rates. There is only limited potential to improve therapy by further time-intensifications and increasing number of cycles. Results are robust against the uncertainty of our parameter estimates.

Many more predictions are possible, especially with respect to stratified therapies in dependence on patient characteristics. We aim to elaborate this issue and possible clinical consequences in the near future. However, translation of model insights into clinical practice is challenging since there are no well established correlates of model parameters with clinical data. One can only speculate at the moment whether for example LDH is a surrogate marker of tumour growth *α* and that stage of disease is a surrogate marker of tumour size *T*_diag_. We also have to acknowledge that we do not have a surrogate marker for immunogenicity since detailed immune statuses of patients are hardly available. A model extension to account for immunotherapy based on the CD20+ antibody Rituximab is also a work in progress.
